# Lower patella–patellar tendon angle is associated with increased odds of patellofemoral osteoarthritis progression in ACL revision patients: A case control study

**DOI:** 10.1002/jeo2.70931

**Published:** 2026-10-05

**Authors:** Domenico Franco, Chilan B. G. Leite, Nicole Krabb, Sebastian Schmidt, Giovanna Medina, Eric M. Berkson, Peter D. Asnis, Lars C. Richardson, Elizabeth G. Matzkin, Cale Jacobs, Christian Lattermann

**Affiliations:** ^1^ Department of Orthopedic Surgery Harvard Medical School, Brigham and Women's Hospital Boston Massachusetts USA; ^2^ Department of Orthopaedic and Trauma Surgery, Medical Faculty Mannheim, University Medical Centre Mannheim University of Heidelberg Mannheim Germany; ^3^ Department of Orthopaedic Surgery Harvard Medical School, Massachusetts General Hospital Boston Massachusetts USA

**Keywords:** anterior cruciate ligament reconstruction, patellofemoral alignment, patellofemoral osteoarthritis, patella‐patellar tendon angle, risk factors

## Abstract

**Purpose:**

To investigate whether a lower preoperative patella–patellar tendon angle (P‐PTA) is associated with progression of patellofemoral osteoarthritis (PFOA) in patients undergoing anterior cruciate ligament (ACL) revision surgery. We hypothesised that patients with lower preoperative P‐PTA would have higher odds of PFOA progression.

**Methods:**

A database search (2015–2023) included patients who underwent ACL revision surgery with magnetic resonance imaging (MRI) available at both initial and revision ACL injuries. Inclusion criteria were primary arthroscopic anterior cruciate ligament reconstruction without cartilage restoration procedures and ≥2 years between ACL injuries. Demographic data, graft type, patellofemoral joint measurements (P‐PTA, Sagittal tibial tubercle‐trochlear groove distance, Insall–Salvati ratio) and PFOA progression were collected at both time points. PFOA progression was defined using MRI osteoarthritis knee score (MOAKS) criteria.

**Results:**

Sixty‐two knees in 61 patients met the inclusion criteria (mean age at primary injury 30.60 ± 12.30 years; body mass index 26.70 ± 4.40 kg/m^2^; mean interval between injuries 4.77 ± 3.04 years). Twenty‐two knees (35.50%) had no PFOA progression, and 40 (64.50%) had progression. Preoperative P‐PTA was significantly lower in the progression group (141.90 ± 4.60°) than in the no‐progression group (144.80 ± 5.70°; *p* = 0.044); this difference persisted at the second injury (142.30 ± 4.90° vs. 145.60 ± 6.60°; *p* = 0.043). Age (*p* = 0.02) and graft type (*p* = 0.015) also differed between groups. Insall–Salvati and sTT‐TG at the first injury did not differ between groups. Multivariable logistic regression identified lower preoperative P‐PTA as an independent predictor of progression (OR: 1.15 per 1° decrease; 95% CI: 1.02–1.30; *p* = 0.022). P‐PTA alone showed limited discrimination (AUC: 0.66, 95% CI: 0.50–0.80), whereas the multivariable model showed higher apparent discrimination (AUC: 0.79, 95% CI: 0.68–0.90). Both receiver operating characteristic analyses were derived and evaluated in the same cohort, and no independent validation was performed.

**Conclusion:**

In this ACL revision cohort, a lower preoperative P‐PTA was independently associated with MOAKS‐defined PFOA progression. Each 1° decrease in P‐PTA was associated with 15% higher odds of progression. However, the mean between‐group difference was smaller than the MDC95, limiting individual‐level interpretation of P‐PTA measurements.

**Level of Evidence:**

Level III.

**Abbreviations**
ACL: anterior cruciate ligament
ACL‐R: anterior cruciate ligament reconstruction
AG: allograft
ALR: anterolateral ligament reconstruction
BMI: body mass index
BPTB: bone‐patellar tendon‐bone autograft
CI: confidence interval
FU: follow‐up
HT: hamstring autograft
ICC: intraclass correlation coefficient
IRB: institutional review board
IS: Insall–Salvati ratio
LET: lateral extra‐articular tenodesis
MOAKS: MRI osteoarthritis knee score
MRI: magnetic resonance imaging
OA: osteoarthritis
OR: odds ratio
PFJ: patellofemoral joint
PFOA: patellofemoral osteoarthritis
PTOA: posttraumatic osteoarthritis
P‐PTA: patella‐patellar tendon angle
SD: standard deviation
STROBE: strengthening the reporting of observational studies in epidemiology
sTT‐TG: sagittal tibial tubercle‐trochlear groove distance

## INTRODUCTION

Anterior cruciate ligament reconstruction (ACL‐R) has become the gold standard treatment for ACL injuries in active populations, consistently demonstrating excellent functional outcomes and high patient satisfaction rates [2, 6]. However, despite these favourable results, the patients remain at substantial risk for developing posttraumatic osteoarthritis (PTOA) in the years following ACL‐R [20]. Prospective longitudinal studies have reported PTOA incidence rates ranging from 37% to 40% at 15 years, regardless of the surgical technique employed [14]. This finding is particularly concerning given that ACL injuries predominantly affect young, physically active individuals with high functional demands and long life expectancy [2, 6].

Among the articular compartments susceptible to PTOA development, the patellofemoral joint (PFJ) has garnered increasing clinical attention in recent years. Current evidence demonstrates that patellofemoral osteoarthritis (PFOA) occurs at rates equivalent to those observed in the tibiofemoral compartment following ACL‐R, with prevalence rates reaching 38%–75% at medium‐to‐long‐term follow‐up [4, 23]. While several factors have been discussed in PFOA development following ACL‐R, including advanced age at time of reconstruction, the graft used, meniscal status and the altered PFJ loading, a comprehensive understanding of imaging‐based risk factors for PFOA in ACL‐R patients remains incomplete [13, 23, 30].

Magnetic resonance imaging (MRI) is one of the most reliable image‐based assessments for detecting the early stages of osteoarthritis (OA). The MRI osteoarthritis knee score (MOAKS) is a validated semi‐quantitative scoring system for longitudinal assessment of knee OA changes, demonstrating excellent inter and intraobserver reproducibility [9].

Recent research has increasingly focused on sagittal PFJ alignment to predict chondropathy [5, 11, 27]. The patella–patellar tendon angle (P‐PTA), which measures the angle between the line connecting the superior and inferior poles of the patella and the line along the patellar tendon from the inferior pole of the patella to the tibial tuberosity [5], was recently associated with PFJ chondral lesions [5, 11, 27]. In fact, patients with patellar chondropathy exhibit significantly lower P‐PTA values compared to healthy controls, suggesting a potential role of this parameter in PFJ pathology [5, 11, 27]. However, the literature remains limited on the potential role of P‐PTA as an associated factor for PFOA progression following ACL‐R, which may reflect altered patellofemoral kinematics. In this regard, P‐PTA has only been considered in cross‐sectional studies and for anterior knee pain conditions or chondropathy. No previous studies have highlighted the potential relationship between P‐PTA and PFOA progression.

The purpose of this study was to compare preoperative P‐PTA between patients who did and did not develop PFOA progression after primary ACL‐R, within a cohort that later underwent ACL revision and had paired MRI at the index and second injuries. We hypothesised that patients with lower preoperative P‐PTA would have higher odds of PFOA progression.

## METHODS

This retrospective case‐control study was approved and conducted at the academic institution of Mass General Brigham, Brigham and Women's Hospital (IRB 2018P001905). A comprehensive database search from 2015 to 2023 was performed to identify patients who underwent ACL revision surgery and had MRI scans available at the time of both their initial ACL injury and second injury leading to ACL revision.

Inclusion criteria comprised patients who had undergone primary arthroscopic ACL‐R with no previous or concurrent cartilage restoration surgeries such as microfracture, chondrocyte implantation or osteochondral grafting. A minimum of 2 years between the first and second injuries was also required to ensure adequate follow‐up for PFOA progression. The unit of analysis was the knee. One patient contributed two knees (bilateral ACL injury and ACL revision), and both knees were retained.

Exclusion criteria encompassed patients with incomplete or suboptimal MRI data, including images affected by motion artifacts. Patients with prior or concurrent knee surgeries other than the primary ACL‐R in association with meniscectomy or repair, such as meniscal transplantation, multi‐ligament reconstruction or osteotomies, were also excluded. Furthermore, patients with concomitant chondral injuries at the time of ACL revision surgery requiring cartilage interventions were excluded to avoid confounding the assessment of PFOA.

Demographic and clinical data were extracted from electronic medical records following the strengthening the reporting of observational studies in epidemiology (STROBE) guidelines. Collected variables included age at primary ACL‐R, sex, body mass index (BMI, kg/m^2^), side, activity level at the time of injury (categorised as active with specification of sports or sedentary), graft type used in the primary ACL‐R (bone patellar tendon bone autograft [BPTB], hamstring autograft [HT] or allograft [AG]), concomitant procedures performed during primary and revision surgeries (such as meniscectomy, meniscal repair, lateral extra‐articular tenodesis [LET] or anterolateral ligament reconstruction [ALR]), smoking status (never/former/current), medical comorbidities and preoperative mental health conditions (depression, anxiety or mood disorders). The follow‐up period was defined as the time interval between the primary and second ACL injuries (years), calculated from the dates of the respective preoperative MRI scans.

### PFJ measurements

All MRI examinations were performed using 1.5 T or 3 T scanners with standardised knee protocols, including T1‐weighted, T2‐weighted fat‐suppressed and proton density sequences in sagittal, coronal and axial planes. PFJ measurements were carried out at both first and second injury time points. They included: (1) P‐PTA, measured as the angle defined by a line drawn from the upper pole to the lower pole of the patella and a line from the lower patellar pole to the midline of the tibial tubercle in the sagittal section where the patellar tendon and upper and lower poles of the patella are aligned (Figure 1) [5], (2) Sagittal tibial tubercle‐trochlear groove distance (sTT‐TG), measured in the sagittal plane as the anteroposterior distance between the tibial tubercle and the trochlear groove. The first point is identified at the deepest point on the anterior surface of the trochlear groove at the subchondral layer, and the second is marked at the anterior aspect of the tibial tubercle. A line is then drawn between these points, perpendicular to the posterior condylar axis, and the distance between these two lines represents the sTT‐TG (Figure 2) [12] and (3) Insall–Salvati ratio (IS), traditionally calculated as the ratio of patellar tendon length to patellar height [28].

**Figure 1:**
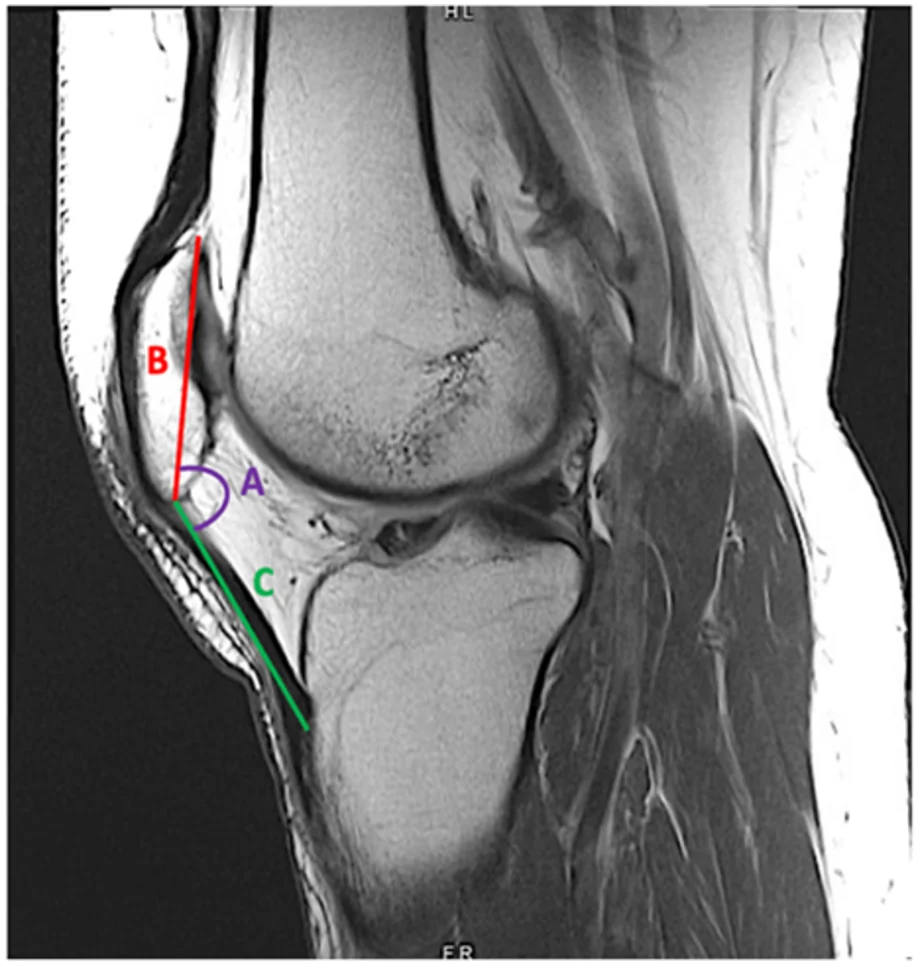
MRI image shows the patella–patellar tendon angle (P‐PTA) measurement. P‐PTA is defined as an angle (A) formed by the red line drawn from the upper to the lower pole of the patella (B) and the green line from the lower patellar pole to the middle of the tibial tubercle on the sagittal section, where the patellar tendon and the upper and lower pole of the patella are aligned (C). MRI, magnetic resonance imaging.

**Figure 2:**
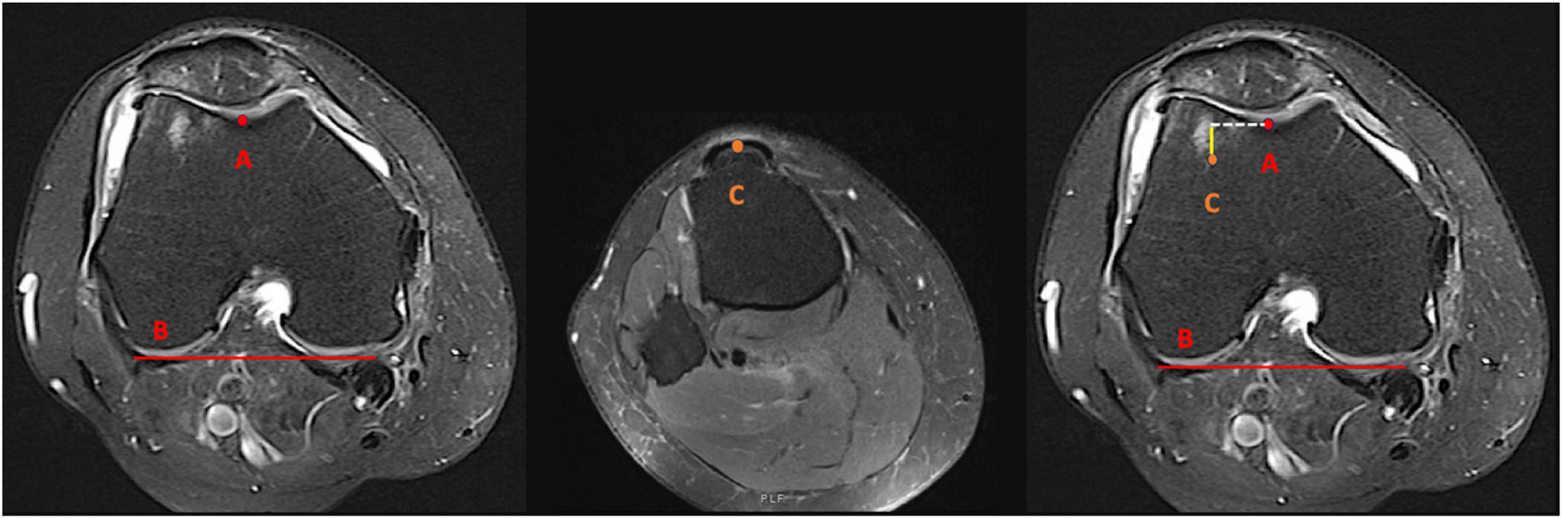
The MRI image displays the sagittal tibia tubercle–trochlear groove (sTT‐TG) measurement. First, the deepest part of the trochlear groove is marked (A), and the posterior condylar axis is drawn for orientation (B). Then, the base of the tibial tubercle is identified (C), and the sTT‐TG distance (yellow line) is measured in millimetres between the projection of point C (perpendicular to the posterior condylar axis) and the line parallel to the posterior condylar axis that intersects point B (white dotted line). MRI, magnetic resonance imaging.

The measurements were performed independently by two fellowship‐trained sports medicine orthopaedic surgeons who were not involved in patient care. Interobserver reliability was evaluated in 21 randomly selected cases. Intraobserver reliability was assessed in a subset of 20 cases by having one reader repeat the imaging measurements in a separate reading session, blinded to the initial measurements. For continuous measurements, interobserver reliability was assessed using a two‐way random‐effects, absolute‐agreement, single‐measure intraclass correlation coefficient (ICC), whereas intraobserver reliability was assessed using a two‐way mixed‐effects, absolute‐agreement, single‐measure ICC. ICC values were interpreted as <0.50, poor; 0.50–0.75, moderate; 0.75–0.90, good; and >0.90, excellent. Because MOAKS progression was dichotomised (0 = no progression; 1 = progression), interobserver agreement for this outcome was assessed using unweighted Cohen's *κ* and observed percentage agreement rather than ICC.

### Patellofemoral OA assessment

The PFOA progression was the primary outcome, assessed using the MOAKS score definition of OA progression. This validated scoring system was used to grade PFJ, including cartilage morphology (size and depth of defects) and osteophytes (size and location). Subregions evaluated included the medial and lateral patella, medial and lateral trochlea and patellar sagittal osteophytes.

PFOA progression was defined according to established criteria as: (1) an increase in the percentage of full‐thickness cartilage loss, or (2) an increase in the size of any cartilage loss when the percentage of full‐thickness cartilage loss remained unchanged, or (3) an increase in the score for an osteophyte scored 2 at baseline or (4) a score of 2 at follow‐up for an osteophyte with a score less than 2 at baseline [21].

Knees were categorised into two groups: those without PFOA progression (MOAKS progression = 0) and those with PFOA progression (MOAKS progression = 1).

### Statistical analysis

All statistical analyses were performed using R software version 4.4.1 (R Foundation for Statistical Computing) and Prism 10, Version 10.4.2 (GraphPad Software). The Shapiro–Wilk test was used to assess the normality of continuous variables in both groups. Continuous variables were presented as mean ± standard deviation (SD) and were compared using *t*‐test or Mann–Whitney *U*‐test (respectively for normally or nonnormally distributed data) and with paired analysis when allowed. Categorical variables were summarised as frequencies and percentages, and were analysed using the Chi‐square test or Fisher's exact test as appropriate.

Reliability estimates were reported with 95% confidence intervals (CI). ICC CIs were derived using the corresponding F distribution, and the 95% CI for Cohen's *κ* was calculated using the large‐sample Wald method. For first‐injury P‐PTA, the standard error of measurement (SEM) was calculated as SD × √(1 − ICC), and the 95% minimal detectable change (MDC95) as 1.96 × √2 × SEM. Pairwise correlations among P‐PTA, sTT‐TG and IS ratio were assessed at each MRI time point using Pearson or Spearman coefficients, according to variable distributions.

The association between first‐injury P‐PTA and PFOA progression was assessed using logistic regression. The primary multivariable model included first‐injury P‐PTA, age at primary injury and primary graft type. These covariates were selected based on clinical relevance while limiting model complexity given the sample size. To evaluate the robustness of the P‐PTA association, exploratory sensitivity analyses were performed by separately adding sex, meniscectomy at primary ACL‐R, preoperative sTT‐TG and IS ratio to the primary model. Postoperative laxity, quadriceps strength and rehabilitation protocol were not consistently available for multivariable adjustment. Results of sensitivity models were interpreted as exploratory.

Receiver operating characteristic (ROC) analysis was performed to quantify the discriminative power of PFOA progression. We constructed ROC curves for preoperative P‐PTA alone and predicted probabilities from the primary multivariable logistic model (preoperative P‐PTA, age and graft type). The area under the curve (AUC) was reported with 95% CIs. For each ROC curve, the optimal cutoff was defined using the Youden index [22].

## RESULTS

A total of 62 knees in 61 patients were included; one patient contributed both knees (Figure 3). The cohort comprised 40 female knees (64.5%) and 22 male knees (35.5%), with a mean age of 30.58 ± 12.29 years. Mean BMI was 26.65 ± 4.40 kg/m^2^ among 61 knees with available BMI, and mean MRI‐to‐MRI follow‐up was 4.77 ± 3.04 years.

**Figure 3:**
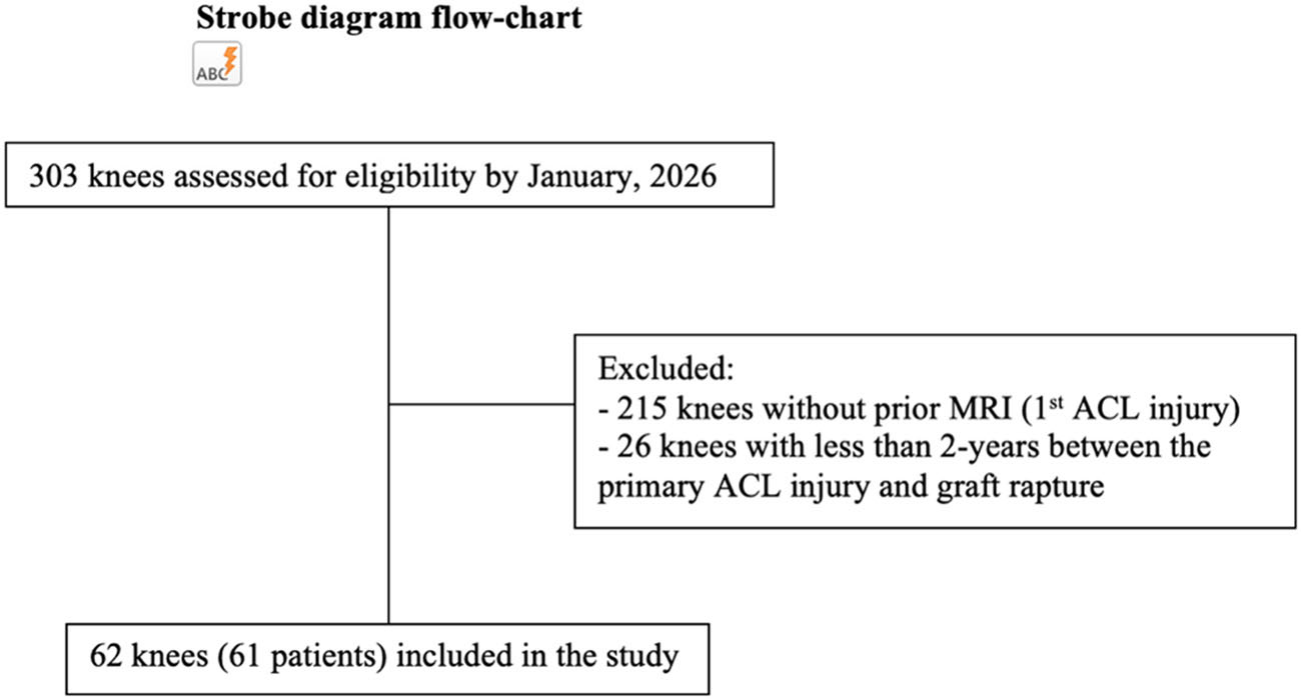
STROBE flow diagram of study selection. Of 303 knees assessed for eligibility, 215 were excluded because a prior MRI at the primary ACL injury was unavailable and 26 because less than 2 years elapsed between the primary ACL injury and graft rupture. The final cohort included 62 knees from 61 patients with paired MRI examinations at the primary and revision ACL injuries. ACL, anterior cruciate ligament; MRI, magnetic resonance imaging; STROBE, strengthening the reporting of observational studies in epidemiology.

Knees were stratified as 22 (35.5%) without and 40 (64.5%) with MOAKS‐defined PFOA progression. The progression group was older (33.18 ± 12.03 vs. 25.86 ± 11.56 years; *p* = 0.023). BMI and follow‐up did not differ significantly (*p* = 0.259 and *p* = 0.751, respectively) (Table 1).

**Table 1:** Demographic and clinical characteristics of the 62 knees included in the study, stratified according to MOAKS‐defined PFOA progression.

Variable	Overall (*n* = 62)	No PFOA Progression (*n* = 22)	PFOA Progression (*n* = 40)	*p*‐Value
Age at first injury, years	30.58 ± 12.29	25.86 ± 11.56	33.18 ± 12.03	0.02
BMI (kg/m^2^)	26.65 ± 4.40	25.78 ± 4.12	27.10 ± 4.52	0.26
MRIs follow‐up (years)	4.77 ± 3.04	4.95 ± 3.55	4.68 ± 2.76	0.75
Primary graft type, *n* (%)				0.02
BPTB autograft	26 (41.9)	7 (31.8)	19 (47.5)	
Hamstring autograft	15 (24.2)	10 (45.5)	5 (12.5)	
Allograft	21 (33.9)	5 (22.7)	16 (40.0)	
Sex, *n* (%)				0.14
Female	40 (64.5)	17 (77.3)	23 (57.5)	
Male	22 (35.5)	5 (22.7)	17 (42.5)	
Side, *n* (%)				0.38
Left	30 (48.4)	9 (40.9)	21 (52.5)	
Right	32 (51.6)	13 (59.1)	19 (47.5)	
Activity level, *n* (%)				0.23
Active	55 (88.7)	18 (81.8)	37 (92.5)	
Passive	6 (9.7)	3 (13.6)	3 (7.5)	
N/A	1 (1.6)	1 (4.5)	0 (0.0)	
Smoking status, *n* (%)				0.20
Never	49 (79.0)	19 (86.4)	30 (75.0)	
Former	11 (17.7)	3 (13.6)	8 (20.0)	
Current	2 (3.2)	0 (0.0)	2 (5.0)	
Preoperative mental status, *n* (%)				0.79
Yes	18 (29.0)	7 (31.8)	11 (27.5)	
No	44 (71.0)	15 (68.2)	29 (72.5)	
Medical comorbidities, *n* (%)				0.43
Yes	30 (48.4)	9 (40.9)	21 (52.5)	
No	32 (51.6)	13 (59.1)	19 (47.5)	

*Note*: Continuous variables are presented as mean ± SD and categorical variables as *n* (%).

Abbreviations: ACL, anterior cruciate ligament; BMI, body mass index; BPTB, bone–patellar tendon–bone; HT, hamstring tendon; MOAKS, MRI osteoarthritis knee score; PFOA, patellofemoral osteoarthritis.

Concomitant procedures at both operations are summarised in Table 2. No individual procedure differed significantly between progression groups. Primary graft distribution differed between groups (*p* = 0.02). In knees without progression, grafts were BPTB in 7 (31.8%), HT in 10 (45.5%) and AG in 5 (22.7%), whereas corresponding counts in knees with progression were 19 (47.5%), 5 (12.5%) and 16 (40.0%), respectively.

**Table 2:** Concomitant surgical procedures performed at primary ACL reconstruction and ACL revision, stratified according to MOAKS‐defined PFOA progression.

Concomitant procedure	Overall (*n* = 62)	No PFOA progression (*n* = 22)	PFOA progression (*n* = 40)	*p*‐Value
Primary ACL‐R				
Meniscectomy	14 (22.6)	2 (9.1)	12 (30.0)	0.11
Meniscal repair	6 (9.7)	2 (9.1)	4 (10.0)	1.00
Root repair	1 (1.6)	0 (0.0)	1 (2.5)	1.00
LET/ALR	1 (1.6)	0 (0.0)	1 (2.5)	1.00
ACL revision				
Meniscectomy	27 (43.5)	9 (40.9)	18 (45.0)	0.80
Meniscal repair	9 (14.5)	3 (13.6)	6 (15.0)	1.00
Root repair	4 (6.5)	1 (4.5)	3 (7.5)	1.00
LET/ALR	23 (37.1)	6 (27.3)	17 (42.5)	0.28

*Note*: Values are presented as *n* (%).

Abbreviations: ACL‐R, anterior cruciate ligament reconstruction; ALR, anterolateral ligament reconstruction; LET, lateral extra‐articular tenodesis; MOAKS, MRI osteoarthritis knee score; PFOA, patellofemoral osteoarthritis.

P‐PTA measurements at the time of the first injury demonstrated significant differences between groups (*p* = 0.04). Knees without PFOA progression had a mean P‐PTA of 144.80° ± 5.70°, while those with PFOA progression had a significantly lower mean P‐PTA of 141.90° ± 4.60°. Moreover, P‐PTA measurements remained significantly different at the time of the second injury (no PFOA group had 145.6° ± 6.6° compared to the progression group with 142.3° ± 4.9°, *p* = 0.04). At the first MRI, neither sTT‐TG (*p* = 0.82) nor IS ratio (*p* = 0.54) differed between progression groups. Similarly, no between‐group differences were observed at the second MRI for sTT‐TG (*p* = 0.74) or IS ratio (*p* = 0.29).

Across all 62 knees, P‐PTA changed from 142.90° ± 5.17° to 143.45° ± 5.74° (mean change 0.55°, 95% CI: −0.69 to 1.79; *p* = 0.38), and sTT‐TG changed from 1.06 ± 5.26 mm to 1.09 ± 5.16 mm (mean change 0.04 mm, 95% CI: −0.04 to 0.12; *p* = 0.32). The IS ratio decreased from 0.99 ± 0.16 to 0.91 ± 0.17 (mean change −0.08, 95% CI: −0.113 to −0.05; *p* < 0.001). By primary graft type, mean IS change was −0.13 (95% CI: −0.17 to −0.08; *p* < 0.001) for BPTB, −0.05 (95% CI: −0.09 to 0.003; *p* = 0.07) for HT, and −0.06 (95% CI: −0.12 to 0.003; *p* = 0.06) for AG; the between‐graft comparison of mean change was borderline (*p* = 0.05). Group‐specific paired estimates are reported in Table 3.

**Table 3:** Patellofemoral MRI measurements at the first and second ACL injuries, stratified according to MOAKS‐defined PFOA progression.

Measure	MRI first injury	MRI second injury	Mean change (95% CI)	*p*‐Value
P‐PTA (degrees ± SD)				
‐MOAKS 0	144.82 ± 5.73	145.64 ± 6.63	0.82 (− 1.49 to 3.13)	0.47
‐MOAKS 1	141.85 ± 4.57	142.25 ± 4.87	0.40 (− 1.12 to 1.92)	0.60
sTT‐TG (mm ± SD)				
‐MOAKS 0–MOAKS 1	0.84 ± 5.941.18 ± 4.92	0.88 ± 5.841.21 ± 4.83	0.05 (− 0.12 to 0.21)0.03 (− 0.05 to 0.12)	0.560.42
IS (value ± SD)				
‐MOAKS 0	0.97 ± 0.18	0.88 ± 0.16	−0.095 (− 0.155 to	0.003
‐MOAKS 1	1.00 ± 0.14	0.93 ± 0.17	0.036) −0.076 (− 0.112 to −0.040)	< 0.001

*Note*: Values at each MRI are presented as mean ± SD; mean changes are reported with 95% CIs. *p*‐Values are from paired *t*‐tests. MOAKS 0 indicates no PFOA progression and MOAKS 1 indicates PFOA progression.

Abbreviations: CI, confidence interval; IS, Insall–Salvati; MOAKS, MRI osteoarthritis knee score; MRI, magnetic resonance imaging; PFOA, patellofemoral osteoarthritis; P‐PTA, patella–patellar tendon angle; SD, standard deviation; sTT‐TG, sagittal tibial tubercle–trochlear groove distance.

At the first MRI, P‐PTA correlated inversely with IS (*r* = −0.38, 95% CI: −0.57 to −0.14; *p* = 0.003) and showed a weak positive correlation with sTT‐TG that did not reach statistical significance (*r* = 0.24, 95% CI: −0.01 to 0.46; *p* = 0.06). The sTT‐TG correlated inversely with IS (*r* = −0.31, 95% CI: −0.52 to −0.07; *p* = 0.01). At the second MRI, P‐PTA was not correlated with sTT‐TG (*r* = −0.03; *p* = 0.80) or IS (*r* = −0.16; *p* = 0.21), whereas sTT‐TG remained inversely correlated with IS (*r* = −0.33, 95% CI: −0.53 to −0.08; *p* = 0.009).

Inter and intraobserver reliability ranged from moderate to excellent (Table 4). First‐injury P‐PTA interobserver reliability was moderate (ICC = 0.70, 95% CI: 0.40–0.88). Using the cohort SD of 5.17°, the SEM was 2.83 and the MDC95 was 7.85°. Intraobserver reliability was good for P‐PTA at the first injury (ICC = 0.90, 95% CI: 0.76–0.96) and at the second injury (ICC = 0.87, 95% CI: 0.70–0.95), excellent for sTT‐TG at the first injury (ICC = 0.99, 95% CI: 0.98–1.00) and moderate for the IS ratio at the first injury (ICC = 0.74, 95% CI: 0.46–0.89). For MOAKS progression, the readers agreed in 19 of 21 cases (90.5%), and Cohen's *κ* was 0.80 (95% CI: 0.53–1.00), indicating substantial chance‐corrected agreement.

**Table 4:** Interobserver and intraobserver reliability of continuous MRI measurements in the reliability subset.

Measure	Interobserver ICC (95% CI)	Intraobserver ICC (95% CI)
P‐PTA (1’ injury time)	0.70 (0.40–0.88)	0.90 (0.76–0.96)
P‐PTA (2’ injury time)	0.76 (0.41–0.92)	0.87 (0.70–0.95)
sTT‐TG (1’ injury time)	0.97 (0.92–0.99)	0.99 (0.98–1.00)
Is (1’ injury time)	0.80 (0.55–0.91)	0.74 (0.46‐0.89)

*Note*: ICCs are reported with 95% CIs and values were interpreted as <0.50, poor; 0.50–0.75, moderate; 0.75–0.90, good; and >0.90, excellent.

Abbreviations: CI, confidence interval; ICC, intraclass correlation coefficient; IS, Insall–Salvati; P‐PTA, patella–patellar tendon angle; sTT‐TG, sagittal tibial tubercle–trochlear groove distance.

In the sensitivity model additionally adjusted for sex, the association between lower first‐injury P‐PTA and PFOA progression remained significant (OR 1.16 per 1° decrease, 95% CI: 1.02–1.32; *p* = 0.02). Sex was not independently associated with progression (female vs. male: OR 0.70, 95% CI: 0.18–2.74; *p* = 0.61).

### Logistic regression analysis

A multivariable regression model was constructed to assess the independent association between P‐PTA at the first injury and PFOA progression, adjusted for age and graft type as predictor variables. P‐PTA was independently associated with PFOA progression (*p* = 0.02). For each one‐degree decrease in P‐PTA, the odds of developing PFOA increased by 15.0% (OR = 1.15, 95% CI: 1.02–1.30). Age and graft type did not reach statistical significance in the multivariable model (Age: *p* = 0.11; HT autografts to AGs: *p* = 0.19; BPTB autografts to AGs: *p* = 0.62; HT to BPTB autografts: *p* = 0.20).

In sensitivity analyses, the association between lower preoperative P‐PTA and PFOA progression was unchanged after separate addition of meniscectomy (OR: 1.15, 95% CI: 1.02–1.30, *p* = 0.02) or sTT‐TG (OR: 1.17, 95% CI: 1.03–1.33, *p* = 0.01). Neither meniscectomy nor sTT‐TG was independently associated with progression in these models (*p* = 0.37 and *p* = 0.30, respectively).

The association also remained significant after additional adjustment for the preoperative IS ratio (OR: 1.15 per 1° decrease in P‐PTA, 95% CI: 1.01–1.31; *p* = 0.030). The IS ratio was not independently associated with PFOA progression (*p* = 0.98).

### Receiver‐operating characteristic (ROC) analysis

Preoperative P‐PTA alone showed limited discrimination for PFOA progression (AUC: 0.66, 95% CI: 0.50–0.80) (Figure 4). The Youden‐optimal cutoff was 142°, yielding a sensitivity of 65% and a specificity of 68%. Predicted probabilities from the full model (P‐PTA, age and graft type) showed higher apparent discrimination (AUC: 0.79, 95% CI: 0.68–0.90). The Youden‐optimal probability cutoff was 0.69 (sensitivity 75%, specificity 73%). Both ROC curves were derived and evaluated in the same cohort, and no independent validation was performed.

**Figure 4:**
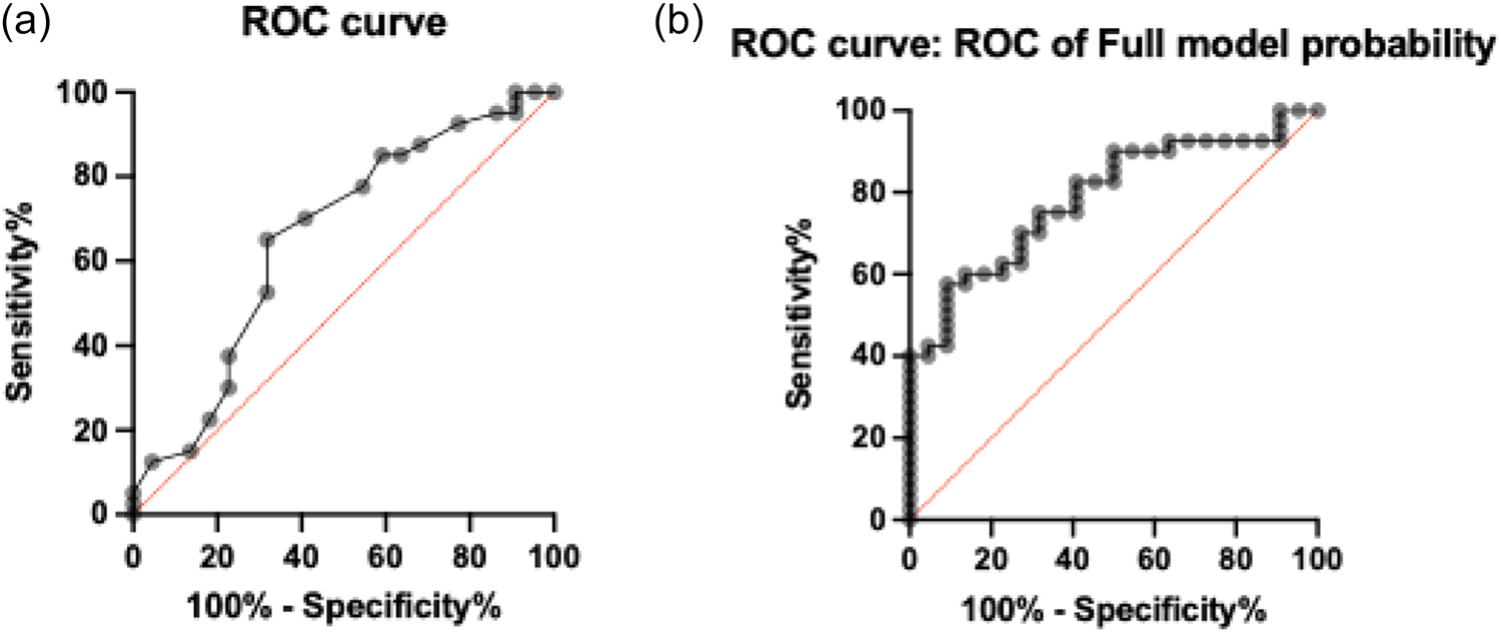
(a) Receiver‐operating characteristic (ROC) curve for preoperative patella–patellar tendon angle (P‐PTA) in discriminating subsequent MOAKS‐defined patellofemoral osteoarthritis (PFOA) progression in the ACL revision cohort (*n* = 62). The area under the curve was 0.66 (95% confidence interval [CI]: 0.50–0.80). The diagonal dashed line represents no discriminative ability. A lower P‐PTA was associated with a greater odds of PFOA progression. The Youden‐optimal threshold was P‐PTA ≤ 142°, with sensitivity 65% and specificity 68%. (b) Predicted probabilities from the multivariable model, including preoperative P‐PTA, age and graft type, showed higher apparent discrimination (AUC 0.79, 95% CI: 0.68–0.90); the Youden‐optimal probability threshold was 0.69 (sensitivity 75%, specificity 73%). The diagonal reference line indicates no discrimination. Both ROC curves were derived and evaluated in the same cohort, and no independent validation was performed. ACL, ACL‐R, anterior cruciate ligament; AUC, area under the curve; MOAKS, MRI osteoarthritis knee score.

## DISCUSSION

This study demonstrates that a lower P‐PTA at the time of initial ACL injury was independently associated with PFOA progression following ACL‐R. For each one‐degree decrease in P‐PTA, the odds of developing PFOA increased by 15% (*p* = 0.02). The observed mean difference of approximately 3° in P‐PTA between knees with and without PFOA progression (respectively: 141.9° vs. 144.8° at first injury; 142.3° vs. 145.6° at second injury) demonstrates consistency across both injury time points, strengthening the evidence that P‐PTA represents a stable anatomical factor associated with PFOA. However, the between‐group mean difference in first‐injury P‐PTA (2.97°) was smaller than the MDC95 of 7.85°. Therefore, although lower P‐PTA was associated with PFOA progression at the group level, a difference of this magnitude should not be interpreted as exceeding measurement error for an individual knee. Similarly, the odds ratio reflects a model‐based association and should not be interpreted as an individual diagnostic effect. Our findings align with emerging investigations showing that patients with PFJ chondropathy have significantly lower P‐PTA values than healthy controls, with reported mean differences ranging from 2° to 5° [5, 11]. Kim et al. reported an AUC of 0.696 with a P‐PTA cutoff of 138.3° in a different clinical setting [11]. Our internally derived 142° threshold should also be interpreted cautiously. This cutoff was derived and evaluated within the same cohort, yielding only modest sensitivity (65%) and specificity (68%). Furthermore, the 95% CI for the P‐PTA AUC extended to 0.50, indicating relative uncertainty in discriminative performance. Accordingly, the 142° value should be regarded as an exploratory cohort‐specific threshold rather than a clinically validated cutoff. Similarly, the higher apparent discrimination of the multivariable model (AUC 0.79) was derived and evaluated within the same cohort, with no independent validation. The current study extends these observations to the ACL revision population, suggesting that the relationship between P‐PTA and PFJ pathology is crucial in the setting of ACL injury and its surgical intervention, addressing a significant gap in the literature. Additionally, the use of the MOAKS scoring system offers advantages over radiographic OA assessments commonly employed in previous investigations [3, 4, 7, 14, 20, 23]. In fact, MRI provides superior sensitivity for detecting early stages of OA that precede radiographically evident OA, potentially allowing for clear identification of at‐risk patients and the implementation of preventive strategies [1, 15, 17].

The biomechanical rationale for this study result stems from the altered PFJ loading patterns [5, 31]. A reduced P‐PTA theoretically indicates changes in the orientation of the patella relative to the trochlea, which may increase contact pressures and predispose to accelerated cartilage degeneration [5]. Finite element modelling studies have demonstrated that small changes in PFJ can result in significant alterations in contact stress distribution [25]. This mechanism may become particularly relevant in the ACL‐R population, where altered knee kinematics and potential postoperative quadriceps weakness may further compromise PFJ loading [18, 19, 26, 29]. Moreover, the common occurrence of quadriceps inhibition and strength deficits after ACL‐R may be a significant factor in patients with reduced P‐PTA, who may be at a higher a priori risk of accelerated cartilage degeneration. Taken together, these findings suggest that reduced P‐PTA may identify ACL‐R patients with abnormal PFJ loading after surgery, which increases the odds of PFOA.

The overall PFOA progression rate of 64.5% in this cohort falls within the 38%–75% range reported at medium‐ to long‐term follow‐up after ACL‐R [4, 23]. The high rate should be interpreted in the context of a selected revision cohort. Although lower P‐PTA may be useful for odds modelling, this study did not evaluate standardised rehabilitation protocols, quadriceps‐loading strategies or graft‐selection interventions. Therefore, the findings do not support specific rehabilitation or graft recommendations. Prospective studies are required before clinical management is changed.

The pathogenesis of PFOA after ACL‐R is multifactorial and depends not only on PFJ variables but also on demographic factors, graft choice, concomitant intraarticular injuries and postoperative knee stability. In the present cohort, meniscectomy at primary ACL‐R was more frequent in the PFOA progression group than in the no‐progression group (30.0% vs. 9.1%), although the difference was not statistically significant (*p* = 0.11).

The nonsignificant findings for sTT‐TG and IS ratio need careful consideration within the broader context. The sTT‐TG distance has been increasingly recognised as an important parameter, with recent evidence suggesting associations with PFJ contact pressure and cartilage degeneration [12, 16].

At the first MRI, P‐PTA was inversely correlated with IS ratio (*r* = −0.38), indicating that lower P‐PTA was associated with a relatively higher patellar position. Thus, the cross‐sectional relationship between P‐PTA and patellar height in this cohort does not support patella baja as an explanation for lower P‐PTA. Separately, IS ratio decreased significantly between the first and second MRI, particularly in the BPTB subgroup, suggesting that postoperative changes in patellar height may occur over time [8, 10, 24]. These longitudinal changes should be distinguished from the baseline relationship between P‐PTA and IS ratio.

The recent literature has highlighted that PFJ loading patterns are significantly altered following ACL‐R [18, 19, 26, 29]. These alterations may be better captured by parameters that assess the functional relationship between anatomical structures rather than other positional measurements. The P‐PTA, by incorporating both patellar position and tendon orientation, may provide a greater sensitivity to these functional alterations that occur following ACL‐R and could be a valuable measure for the progression of PFOA after ACL‐R. An unresolved question remains as to whether a lower P‐PTA primarily reflects a preoperative morphotype or a secondary change after ACL injury and a failed primary ACL‐R (increased anterior tibial translation). Because P‐PTA incorporates the patella's long axis and the patellar tendon vector, it could be theoretically influenced by patellar height. Establishing causality is beyond the design of this case–control study and will require imaging before primary ACL injury, after ACL‐R or considering the contralateral uninjured knee.

### Limitations

The retrospective nature of this investigation introduces inherent limitations, including potential selection bias and incomplete data capture. The study cohort was derived from patients undergoing ACL revision surgery, which may not be the best representative sample of the broader ACL‐R population. Patients requiring revision surgery may have had more severe initial injuries or suboptimal outcomes from their surgery, potentially amplifying the observed results.

The sample size was not based on an a priori calculation and was limited by the availability of dual MRI in revision cases. Therefore, the sample size may limit the statistical power to detect interactions between variables and may increase the risk of type II errors in secondary analyses.

Regarding the graft type related to PFOA progression, the significant association did not persist in the multivariable analysis. While this may reflect limited power due to sample size constraints, it suggests that anatomical factors, such as P‐PTA, are also fundamental determinants of increased likelihood of PFOA.

The mean follow‐up period of 4.77 years, despite being adequate for detecting early PFOA changes, may not capture the full spectrum of long‐term OA development. Longer follow‐up studies will be necessary to determine whether the relationship between P‐PTA and PFOA persists or evolves over extended time periods.

Finally, unmeasured confounders such as postoperative laxity, quadriceps strength, patient compliance and rehabilitation may have influenced the findings. Sex also warrants consideration because prior work has reported sex‐related P‐PTA differences, and females comprised 64.5% of this cohort (77.3% of knees without progression vs. 57.5% with progression). However, adding sex to the primary model did not materially attenuate the P‐PTA association (adjusted OR 1.16 per 1° decrease), and sex itself was not significant; the wide sex confidence interval nevertheless reflects limited precision. Residual confounding and the selected revision population limit causal and generalisable interpretation.

## CONCLUSION

This study provides evidence that lower P‐PTA was independently associated with PFOA progression in ACL revision patients, with each 1° decrease associated with 15% higher odds of progression. However, the mean between‐group difference was smaller than the MDC95, limiting individual‐level interpretation of P‐PTA measurements.

## AUTHOR CONTRIBUTIONS

*Study conception and design, data analysis, manuscript drafting*: Domenico Franco, Chilan B. G. Leite and Christian Lattermann. *Data interpretation, critical revision, final approval*: All authors.

## FUNDING INFORMATION

The authors have no funding to report.

## CONFLICT OF INTEREST STATEMENT

The authors declare no conflicts of interest.

## ETHICS STATEMENT

Mass General Brigham, Brigham and Women's Hospital, IRB number 2018P001905.

## Data Availability

The data are available from the corresponding author on reasonable request.
